# Mechanisms Underlying Adaptation of Respiratory Network Activity to Modulatory Stimuli in the Mouse Embryo

**DOI:** 10.1155/2016/3905257

**Published:** 2016-04-28

**Authors:** Marc Chevalier, Rafaël De Sa, Laura Cardoit, Muriel Thoby-Brisson

**Affiliations:** Institut de Neurosciences Cognitives et Intégratives d'Aquitaine, CNRS UMR 5287, Université de Bordeaux, 33076 Bordeaux, France

## Abstract

Breathing is a rhythmic behavior that requires organized contractions of respiratory effector muscles. This behavior must adapt to constantly changing conditions in order to ensure homeostasis, proper body oxygenation, and CO_2_/pH regulation. Respiratory rhythmogenesis is controlled by neural networks located in the brainstem. One area considered to be essential for generating the inspiratory phase of the respiratory rhythm is the preBötzinger complex (preBötC). Rhythmogenesis emerges from this network through the interplay between the activation of intrinsic cellular properties (pacemaker properties) and intercellular synaptic connections. Respiratory activity continuously changes under the impact of numerous modulatory substances depending on organismal needs and environmental conditions. The preBötC network has been shown to become active during the last third of gestation. But only little is known regarding the modulation of inspiratory rhythmicity at embryonic stages and even less on a possible role of pacemaker neurons in this functional flexibility during the prenatal period. By combining electrophysiology and calcium imaging performed on embryonic brainstem slice preparations, we provide evidence showing that embryonic inspiratory pacemaker neurons are already intrinsically sensitive to neuromodulation and external conditions (i.e., temperature) affecting respiratory network activity, suggesting a potential role of pacemaker neurons in mediating rhythm adaptation to modulatory stimuli in the embryo.

## 1. Introduction

Breathing is a rhythmic behavior that must be continuously expressed in order to ensure life. However, this vital function must also be constantly adaptable to changing external and internal conditions to satisfy the organism's complex metabolic needs. Thus, the central command for respiration is under the influence of multiple neuromodulatory systems arising from different parts of the central nervous system, which participate in maintaining the efficacy and adaptability of respiratory motor output.

Respiratory function is controlled by neuronal network assemblages located in the brainstem. Several studies have shown that the neural network of the preBötzinger complex (preBötC) that generates inspiration plays a critical role in respiratory rhythm generation [1–5]. This network contains excitatory glutamatergic neurons, a subpopulation of which expresses intrinsic membrane pacemaker properties [6–9]. The activity of the preBötC network is known to be influenced by several biogenic amines and peptides, such as Substance P (SP), opioids, serotonin, acetylcholine, and norepinephrine (for review, see [10]). To adjust neural network operation, by acting on either synaptic strength or cellular properties, neuromodulators can regulate the frequency, regularity, or amplitude of the respiratory motor burst rhythm, as well as the generation of different respiration-related activities (eupnea, sigh, and gasp) [11–18]. The actions of modulators are mediated through the activation of specific receptors whose expression in the preBötC area, at least for the neurokinin 1 receptor NK1R (binding site for SP) and the *μ* opioid peptide receptors *μ*OR, has been demonstrated [11].

The developmental emergence of the rhythmogenic neural circuits responsible for respiration occurs during the last third of gestation. Typical anatomical and functional characteristics of the postnatal preBötC network are detectable from embryonic day (E) 15.5 and E17.5 in mouse and rat, respectively [19–21]. At the time of the onset of a respiratory rhythmogenic capability, it has been shown that activity of the prenatal preBötC network can be influenced by SP and DAMGO (a *μ* opioid receptor agonist) [19, 21]. However, the cellular locus and mechanisms for modulation of the respiratory rhythm by SP and DAMGO at embryonic stages have not yet been investigated. In this context, moreover, the presence of neurons possessing pacemaker properties in the embryonic respiratory network has only been inferred [19, 22], and the potential role of such cells in mediating neuromodulatory influences on the embryonic respiratory rhythm remains completely unknown.

In the present study, therefore, we aimed to investigate whether respiratory pacemaker neurons could be involved in the adaptability of the mouse embryonic respiratory network activity to the influence of modulatory signals. We present results on several such processes affecting the respiratory network activity at prenatal stages. Specifically, we show that the embryonic respiratory rhythm can be modulated by exposure to changes in temperature and also by the peptides SP and DAMGO. These two substances were chosen because they are well known respiration neuromodulators (see above) and also because they exert opposing effects on the postnatal respiratory network, thus allowing the investigation of a large range of activity changes. We find that some inspiratory network neurons identified as pacemaker cells are intrinsically sensitive to both of these modulatory peptides as well as to temperature. The effect on respiratory rhythm cycle frequency observed at the network level under these different extrinsic influences might therefore be mediated, at least in part, through changes specific to pacemaker neuron activity within the embryonic preBötC network.

## 2. Materials and Methods

### 2.1. Animals

All experiments were conducted in accordance with guidelines issued by the European and French National legislations on animal experimentation and have also been approved by the Bordeaux University ethics committee. Pregnant OF1 mice were obtained from our local animal facility, with the presence of a vaginal plug the day after mating being considered as embryonic day (E) 0.5 in gestation. Pregnant females were isolated in a separate cage until their experimental use.

### 2.2. *In Vitro* Slice Preparations

Brainstem transverse slices isolating the preBötC network were obtained from mouse embryos harvested during the developmental period between E16.5 and E18.5. The methods used for preparing slices have been previously described in detail [19, 20]. Briefly, after cervical dislocation of pregnant mice, embryos were isolated and placed at 20°C under continuous oxygenation (by bubbling 95% O_2_/5% CO_2_) in artificial cerebrospinal fluid (aCSF) with the following composition (in mM): 120 NaCl, 8 KCl, 1.26 CaCl_2_, 1.5 MgCl_2_, 21 NaHCO_3_, 0.58 NaH_2_PO_4_, and 30 glucose, pH 7.4. The first step of the dissection consisted of isolating the hindbrain by making a rostral transverse section at the anterior limit of the rhombencephalon and a caudal section at the level of the rostral part of the spinal cord. After isolation, hindbrains were embedded in a low melting point agar block and serially sectioned plane in the transverse in a rostral to caudal direction using a vibratome (Leica). Transverse sections were cut until the axial level of the facial motor nucleus was reached. Past its posterior limit, an additional 250/300 *μ*m thick slice was removed in order to reach the anterior axial level of the 450 *μ*m slice in which the preBötC network was isolated. Slice preparations were then placed in a recording chamber and continuously superfused with oxygenated aCSF maintained at 30°C. The slices were positioned rostral side up and maintained in place by a grid to avoid any movement during optical and electrophysiological recording sessions. We systematically waited for 30 min before starting any recording procedures. The temperature of the aCSF superfusant in the chamber was set using a PTC-10 temperature controller (npi electronic, Tamm, Germany) and a HPT-2 heated perfusion tube (ALA Scientific Instruments, New York, USA).

### 2.3. Electrophysiology

Extracellular recordings of local population activity were made using an aCSF-filled glass macroelectrode fabricated from filamented borosilicate glass tubing (Clark GC 150 F, Harvard Apparatus, France) and positioned at the surface of the slice in the preBötC area on one side. This electrode was connected through a silver wire to a high-gain amplifier (AM System, Carlsborg, USA). The signals were filtered (bandwidth 3 Hz–3 kHz), integrated with a time constant of 100 ms using an electronic filter (NeuroLog System; Digitimer, Hertfordshire, UK), recorded, and stored on a computer via a Digidata 1322 interface (Molecular devices, Sunnyvale, USA) and analyzed offline using PClamp10 software (Molecular Devices).

### 2.4. Calcium Imaging

Slice preparations were incubated in the dark at room temperature for 40 min in oxygenated aCSF containing the cell-permeable calcium indicator dye Calcium Green-1 AM (10 *μ*M; ThermoFisher, France). After this incubation period, preparations were placed in the recording chamber and an additional 30 min period was followed in order to let the preparation recover and stabilize in oxygenated aCSF at 30°C. Fluorescent images were captured from the rostral surface of the slice through a FN1 upright microscope (Nikon) equipped with a 40x objective, a standard epifluorescent illumination system, and a fluorescein filter, coupled to either an Exiblue camera (QImaging, Surrey, Canada) or an Andor camera (Andor Technology Ltd., Belfast, UK) operating with an exposure time of 100 ms in overlapping exposure and readout mode. Images were acquired over periods lasting 120 s and analyzed using customized software developed by Dr. Mellen and Tuong [23]. Neuronal calcium transients extracted automatically using this software were expressed as relative fluorescence changes (Δ*F*/*F*) compared to silent periods.

### 2.5. Pharmacological Treatments and Statistics

The pharmacological cocktail used to synaptically isolate pacemaker neurons contained 20 *μ*M 6-cyano-7-nitroquinoxaline-2,3-dione (CNQX), 10 *μ*M DL-2-amino-5-phosphonovaleric acid (AP5), 1 *μ*M strychnine, and 10 *μ*M bicuculline (all from Sigma).

Substance P (SP, Sigma) and the *μ* opioid receptor agonist D-Ala^2^-*N*-Me-Phe^4^-Glycol^5^-enkephalin (DAMGO, Sigma) were dissolved in aCSF and bath-applied at their final concentration of 0.5 *μ*M and 0.25 *μ*M, respectively. A single modulatory drug was applied per preparation in order to avoid cross effects. Preparations were exposed to a drug for a 15 min period and its effects on respiratory rhythm cycle frequency were analyzed at the end of this period using Clampfit software (Molecular Devices). The reversal of any observed effects was tested for each drug after a 30 min period wash-out during which fresh aCSF was superfused in the recording chamber.

Values are given as mean ± SEM. Student's *t* tests with a Mann-Whitney Rank Sum test or ANOVA tests were used to assess differences (taken to be significant at *P* < 0.05) between values in the presence of the drugs and controls.

### 2.6. Immunostaining

Isolated brainstem preparations were fixed for 3 h in 4% paraformaldehyde, cryoprotected in 20% PBS-sucrose overnight, and then cryosectioned at 30 *μ*m using a cryostat (Leica). Sections were incubated for 1 h30 in 1% BSA and 0.3% Triton X-100, followed by affinity-purified primary antibodies rabbit anti-NK1R (1/10000; Sigma) and goat anti-ChaT (1/100; Millipore) overnight at room temperature. After multiple rinsing in PBS, sections were exposed for 2 h to the secondary antibodies Alexa Fluor 488-conjugated donkey anti-goat IgG (1 : 500; ThermoFisher, France) and Alexa Fluor 568-conjugated donkey anti-rabbit IgG (1 : 500; ThermoFisher). After rinsing with PBS, sections were cover-slipped, mounted in Vectashield, and observed on a Leica microscope. Control experiments in which the primary antibodies were replaced by normal serum were also conducted to ensure lack of labeling and antibody specificity.

## 3. Results

### 3.1. Modulation of the Embryonic Respiratory Rhythm

**Figure 1:**
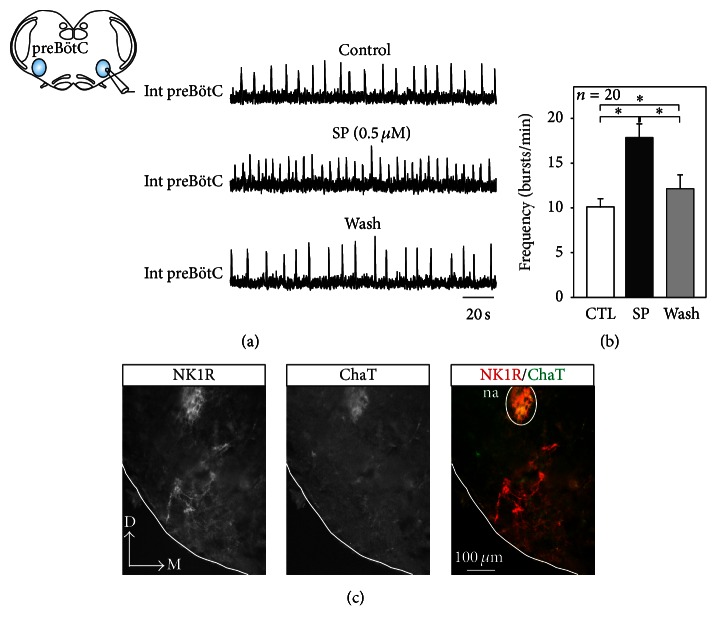
Substance P- (SP-) dependent modulation of the embryonic respiratory rhythm. (a) Schematic of the embryonic transverse brainstem slice isolating the preBötC network with the position of an extracellular recording electrode indicated. Integrated network activity (Int preBötC) recorded in control conditions (top), in the presence of 0.5 *μ*M SP (middle) and after 30 minutes' wash-out (bottom). (b) Pooled data showing mean (± SEM) inspiratory burst frequency obtained from 20 embryonic slices in control conditions (CTL, white bar), after 15 min exposure to 0.5 *μ*M SP (black bar) and after wash-out (gray bar). SP significantly increased the frequency of the spontaneous preBötC network rhythm. This effect was also partially reversible. The asterisk indicates significant difference at *P* < 0.05. (c) Immunodetection of NK1R receptors (red staining) in the preBötC area located ventral to the nucleus ambiguus (na) that was positive to both NK1R (red) and ChaT (green). D: dorsal, M: medial, and na: nucleus ambiguus.

In a first set of experiments, we tested the sensitivity of the embryonic respiratory network to two well-known neuromodulators: SP that exerts an excitatory effect on the postnatal respiratory rhythm and the *μ* opioid peptide agonist DAMGO known to depress newborn respiration [11, 19]. Slice preparations were obtained from embryos between E16.5 and E18.5. Because we could not detect any statistical differences between results obtained at different developmental stages, data were pooled. Pharmacological agents were bath-applied for 15 minutes and changes in cycle frequency of the spontaneous fictive respiratory rhythm were measured by electrophysiological recording of preBötC network activity at the end of the exposure period. As shown in Figures 1(a) and 1(b), 0.5 *μ*M SP induced a significant cycle frequency increase from 10 ± 0.9 bursts/min in control conditions to 17.8 ± 1.5 bursts/min in the presence of SP (*n* = 20). This effect was partially reversible since the mean cycle frequency returned to 12.6 ± 1.7 bursts/min after 30 min wash-out with normal aCSF. Moreover, since SP is known to bind to neurokinin 1 receptors (NK1Rs), we verified that NK1Rs were significantly expressed in the preBötC region of our embryonic preparations. Immunostaining for NK1R revealed that the respiratory network, located ventral to the nucleus ambiguus which is itself identifiable by its choline acetyltransferase (ChaT) and NK1R coimmunoreactivity, was indeed positive for NK1R (Figure 1(c)).

**Figure 2:**
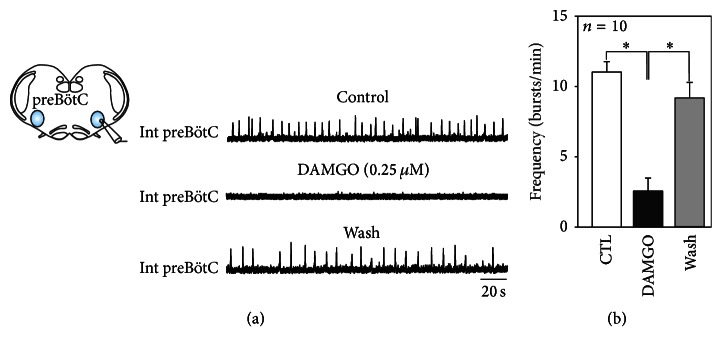
DAMGO-dependent modulation of the embryonic respiratory rhythm. (a) Schematic of an embryonic transverse brainstem slice used for extracellular electrode recording from the isolated preBötC network. Integrated network activity (Int preBötC) recorded in control conditions (top), in the presence of 0.25 *μ*M DAMGO (middle) and after 30 minutes' wash-out (bottom). (b) Pooled data showing mean (± SEM) inspiratory burst frequency obtained from 10 embryonic slices in control conditions (CTL, white bar), after 15 min exposure to 0.25 *μ*M DAMGO (black bar) and after wash-out (gray bar). DAMGO significantly decreased the preBötC rhythm frequency. This effect was reversible. Asterisk indicates *P* < 0.05.

Similarly, we investigated whether the embryonic respiratory rhythm could be modulated by the *μ* opioid receptor agonist DAMGO. Bath application of 0.25 *μ*M DAMGO induced a significant decrease in respiratory cycle frequency from 11 ± 0.7 bursts/min in control conditions to 2.5 ± 0.9 bursts/min under DAMGO (*n* = 10) (Figures 2(a) and 2(b)). Indeed, in almost half of our experiments (*n* = 4), the opioid agonist even caused a complete suppression of the spontaneous respiratory rhythm (see bottom trace in Figure 2(a)). However, in each case, the fictive respiratory frequency returned towards control values conditions (9.1 ± 1.2 bursts/min) when preparations again superfused with normal aCSF (Figure 2(a) bottom trace and gray bar in Figure 2(b)). Altogether these experiments demonstrate that the activity of the respiratory network can be strongly modulated by both exogenous SP and DAMGO at prenatal stages.

### 3.2. Identification of Pacemaker Neurons in the Embryonic Respiratory Network

**Figure 3:**
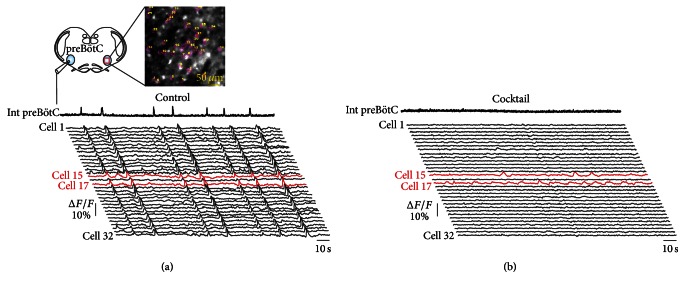
Detection of pacemaker neurons in the embryonic respiratory network. (a) Top: schematic of an embryonic transverse brainstem slice used for extracellular electrode recording from the isolated preBötC network on one side and calcium imaging of the contralateral preBötC network. The fluorescent image is an example where 32 preBötC cells expressed calcium transients simultaneously and in phase with the whole network. Bottom: simultaneous recordings of network electrophysiological activity (Int preBötC, top) and calcium transients (Δ*F*/*F*) in individual cells numbered 1–32 from top to bottom. All selected cells displayed fluorescent changes in phase with inspiratory bursts monitored at the network level. (b) Same recordings after blockade of chemical synapses within the network by exposure to a cocktail of 20 *μ*M CNQX, 10 *μ*M AP5, 10 *μ*M bicuculline, and 1 *μ*M strychnine. The majority of cells fell inactive (black traces), but a few neurons remained capable of generating spontaneous rhythmic fluorescent changes (red traces). These cells were thus identified as preBötC pacemaker neurons.

In order to be able to investigate the role of pacemaker neurons in mediating this modulation of the embryonic respiratory rhythm, we first had to locate this specific cell type within the respiratory network. A pacemaker neuron is characterized by its ability to remain intrinsically capable of spontaneously generating rhythmically organized activity even when synaptically isolated from all other neuronal partners in the network. However, pacemaker neurons are known to comprise a very small proportion (<15%) of the preBötC network [8]. Therefore, to more efficiently detect these cells, we used calcium imaging that allows monitoring simultaneously the activity of a large number of cells in a single preparation. In control conditions, preBötC respiratory neurons were identified by their expression of fluorescence changes in phase with network electrophysiological activity monitored by extracellular recording from the surface of the slice in the contralateral preBötC area (Figure 3(a)). Then, excitatory and inhibitory synapses were blocked by bath application of a cocktail solution containing 20 *μ*M CNQX to block glutamatergic synapses, 10 *μ*M AP5 to block NMDA synapses, 10 *μ*M bicuculline to block GABAergic synapses, and 1 *μ*M strychnine to block glycinergic synapses. Under such conditions, the network fails to generate rhythmically organized activity (see the Int preBötC trace in Figure 3(b); cf. Figure 3(a)) and most of the cells fall inactive (see black traces in Figure 3(b)). In contrast, a few neurons remain rhythmically active (see red traces in Figure 3(b)) and therefore are likely to be constituents of the inspiratory pacemaker cell population in the embryonic preBötC network. Investigating the electrophysiological characteristics of these pacemaker cells is beyond the scope of the present study and will be reported elsewhere (Chevalier et al., in preparation). Here, however, the above population recording approach allowed us to identify a total of 50 isolated pacemaker neurons in 38 preparations, which were subsequently tested for their sensitivity to SP or DAMGO.

### 3.3. Intrinsic Sensitivity of Embryonic Pacemaker Neurons to Substance P

**Figure 4:**
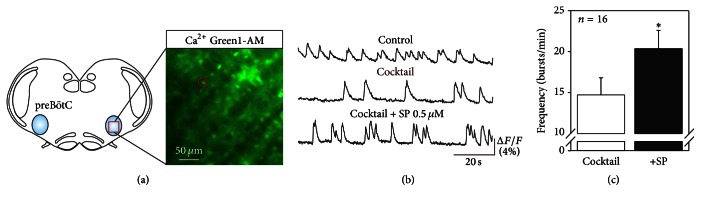
Embryonic respiratory pacemaker neurons are sensitive to SP. (a) Schematic of an embryonic transverse slice showing location of a preBötC network pacemaker neuron monitored with calcium imaging. (b) Traces of calcium transients recorded in the cell in control conditions (top), after synaptic isolation from the other network elements (middle, cocktail) and in the presence of 0.5 *μ*M SP (bottom). After isolation, the frequency of the cell's ongoing rhythmic activity was significantly increased in the presence of SP. (c) Pooled data showing mean frequency (± SEM) of activity expressed by 16 pacemaker neurons in cocktail (white bar) and under the presence of SP (black bar). Asterisk: *P* < 0.05.

With a test population of 24 cells (from 18 preparations) that were coactive with the preBötC network in control conditions and that remained rhythmogenic after synaptic isolation, we investigated their sensitivity to SP by assessing the effects of bath-applied 0.5 *μ*M SP on the frequency of somatic fluorescent variations (Figure 4(a)). As illustrated in Figure 4(b), which shows effects also observed for 16 out of the 24 investigated cells, the frequency of spontaneous fluorescent fluctuations was significantly increased in the presence of SP. The mean frequency increased from 14.7 ± 2 bursts/min in conditions of synaptic blockade to 20.3 ± 2.2 bursts/min (*P* < 0.05, *n* = 16) in the additional presence of SP (Figure 4(c)). We also found 6 pacemaker neurons for which SP application failed to induce any significant changes in their fluorescent signals and 2 further cells that were initially silent after synaptic isolation but became rhythmically active under SP. These latter neurons might correspond to the so-called “conditional” pacemaker neurons that become activated due to the membrane depolarizing effects of SP [13]. Thus, together these results show that a significant proportion of pacemaker neurons within the embryonic respiratory network are intrinsically sensitive to SP, strongly suggesting that they play a major role in the overall frequency increase observed at the network level in the presence of the neuromodulator.

### 3.4. Intrinsic Sensitivity of Embryonic Pacemaker Neurons to DAMGO

**Figure 5:**
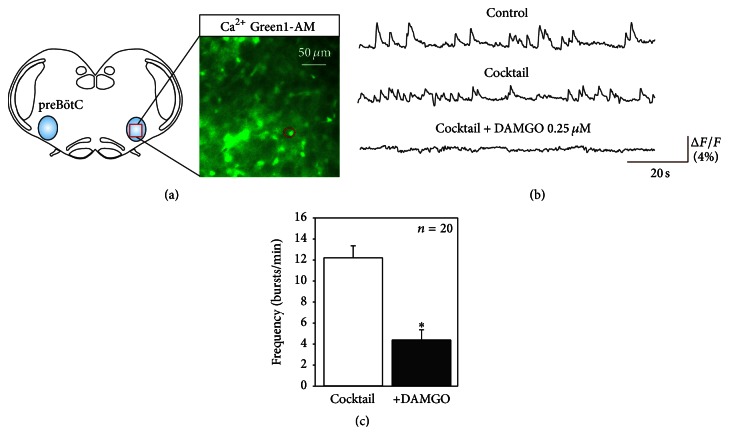
Embryonic respiratory pacemaker neurons are sensitive to DAMGO. (a) Schematic of an embryonic transverse slice showing location of a preBötC pacemaker neuron monitored with calcium imaging. (b) Calcium transients recorded from the cell in control conditions (top), after synaptic isolation (middle, cocktail), and in the presence of 0.25 *μ*M DAMGO (bottom). After isolation, the cell's rhythmic activity was suppressed in the presence of DAMGO. (c) Pooled data showing mean frequency (± SEM) of activity expressed by 20 pacemaker neurons in cocktail (white bar) and under the presence of DAMGO (black bar). Asterisk: *P* < 0.05.

With the 26 remaining pacemaker neurons detected in 20 other preparations by the same isolation procedure, we examined their sensitivity to DAMGO (0.25 *μ*M). Among them, 20 showed a significant decrease in calcium transient frequency in the presence of the *μ* opioid receptor agonist (Figures 5(a) and 5(b)), with the mean cycle frequency declining from 12 ± 1.2 bursts/min in control conditions to 4.3 ± 0.9 bursts/min (*P* < 0.05) under DAMGO (Figure 5(c)). For 6 of these neurons, we even observed a full blockade of their spontaneous activity when exposed to DAMGO. In contrast, the activity of the 6 remaining cells exhibited no detectable modulation by DAMGO. These experiments therefore indicate that a significant subpopulation of preBötC pacemaker neurons are also already inherently sensitive to *μ* opioid peptides at embryonic stages and that these cells are likely to participate in mediating the modulatory effects of the *μ* opioid receptor agonist that we observe on the overall activity of the preBötC network.

### 3.5. Intrinsic Sensitivity of Embryonic Pacemaker Neurons to Temperature

**Figure 6:**
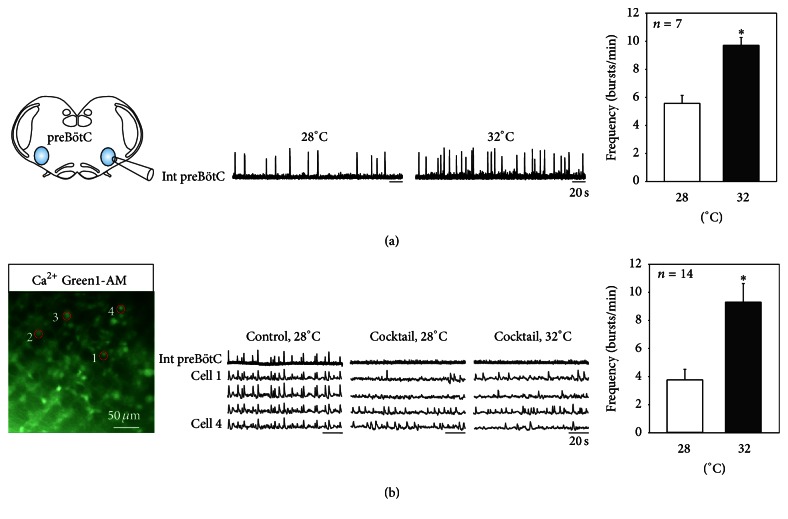
The embryonic respiratory network and respiratory pacemaker neurons respond to temperature changes. (a) Left: schematic of an embryonic transverse slice used for extracellular electrode recording from the isolated preBötC network. Middle: integrated traces of spontaneous network activity recorded at 28°C (left) and at 32°C (right). Right: pooled data showing mean inspiratory burst frequency (± SEM) in 7 slices in control conditions at 28°C (white bar) and at 32°C (black bar). Asterisk: *P* < 0.05. (b) Left: fluorescent image showing location of 4 preBötC network neurons monitored with calcium imaging. Middle: simultaneous recordings of network electrophysiological activity (Int preBötC) and calcium transients in the 4 individual cells in control conditions at 28°C (left), after synaptic isolation in cocktail at 28°C (middle), and in cocktail at 32°C (right). Note that the frequency of spontaneous cyclic activity in isolated cells 1 and 3 increased at 32°C, while that of cell 4 decreased and cell 2 only became active at the elevated temperature. Right: pooled data showing mean frequency (± SEM) of activity expressed by 14 pacemaker neurons under cocktail at 28°C (white bar) and in cocktail at 32°C (black bar). Asterisk: *P* < 0.05.

Another potential source of respiratory activity modulation is external cues, such as temperature. Indeed it is well known that a temperature elevation evokes an increase in respiratory frequency, probably to ensure cooling down the body and returning the temperature to its normal range. Therefore, we investigated first whether the fictive respiratory rhythm at embryonic ages can already be modulated by changes in temperature and, second, if pacemaker neurons might play a role in this temperature-related modulation. We recorded 7 slice preparations in conditions in which the temperature was increased steadily from 28°C up to 32°C, while the frequency of the spontaneous preBötC network rhythm was measured in both conditions (Figure 6(a)). Despite the limited temperature range employed, we observed a significant variation in mean frequency values from 5.6 ± 0.5 bursts/min at 28°C to 9.7 ± 0.5 bursts/min at 32°C in all preparations tested, suggesting that the embryonic respiratory network is indeed very sensitive to temperature changes.

We then tested whether the activity of isolated inspiratory pacemaker neurons is itself affected by changes in external temperature. From 8 slice preparations, we identified 23 pacemaker neurons using calcium imaging and pharmacological blockade of intranetwork synapses as described previously. As illustrated in Figure 6(b), all cells (*n* = 23/23) in this experimental series were rhythmically active in phase with the whole network in control conditions and at a temperature of 28°C. Also, when synaptically isolated at this temperature, most of these pacemaker cells remained rhythmically active at their own inherent frequency (*n* = 19/23), while 4 of them ceased to generate any detectable activity (but see below). Subsequently, increasing the temperature from 28° to 32°C induced four different responses. First, rhythmic activity frequency increased for 14 of the pacemaker neurons from 3.7 ± 0.7 bursts/min at 28°C to 9.3 ± 1.3 bursts/min (see, e.g., cells 1 and 3 in Figure 6(b)). Second, two neurons exhibited an actual frequency decrease, as illustrated for cell 4 in Figure 6(b). Third, 3 neurons were unaffected by the temperature change. Fourth, 4 neurons behaved as conditional pacemakers in that they were inactive at 28°C but began to generate spontaneous fluorescent changes when placed at 32°C (cell 2, Figure 6(b)). Altogether, these results therefore indicate that the response of the respiratory network to temperature elevation is complex at the pacemaker cellular level, although the overall effect on network output is a reliable increase in respiratory rhythm frequency.

## 4. Discussion

This study establishes that the respiratory network in murine embryos is already functionally plastic in that its operation is likely to depend on and adapt to the neuromodulatory milieu and external temperature. In addition, we provide evidence that pacemaker neurons in the preBötC network might be importantly involved in several of the modulatory processes targeting embryonic respiratory activity as indicated by our finding that these pacemaker cells are individually sensitive to SP, DAMGO, and temperature changes. However, it is probable that endogenous pacemaker neurons are not the sole target for neuromodulators within the respiratory network. Indeed it has been shown in the newborn rodent that modulators such as SP and norepinephrine act on both pacemaker and nonpacemaker inspiratory neurons [13, 16]. Moreover, not every pacemaker we recorded was sensitive to SP, DAMGO, or temperature changes, which would be consistent with a type of fail-safe mechanism in which pacemaker neurons are not all involved in a given modulatory function. So even though the role of pacemaker neurons in respiratory rhythmogenesis is still a matter of some debate, it is clear that at least some of them are capable of participating in modulator-derived frequency adjustments of respiratory network activity at embryonic ages.

There are several ways in which the patterned activity of a rhythmogenic neural network can be modulated: by regulating the strength of its constituent neurons' synaptic connections, by modulating their membrane properties, and by altering the size of the active cell population within the network. Because we worked mainly on respiratory neurons in functional synaptic isolation, we could not address the first of these possibilities. Apart from the evidence that our study provides in pinpointing the second of these modulatory processes, we also found indications for the third possibility, namely, a modulation of functional network size. This derived from the finding that some preBötC network cells, which were spontaneously silent after blockade of synaptic connections, became rhythmically active upon exposure to SP or temperature changes. These neurons thus corresponded to the so-called conditional pacemaker neurons for which specific extrinsic conditions are required for their spontaneous bursting to occur [13, 16, 24]. On this basis, therefore, the number of pacemaker neurons within the embryonic respiratory network is not fixed but presumably can be dynamically regulated by neuromodulators or external conditions that control the functional status and thus participation of individual cells in generating network activity. While the use of calcium imaging did not allow us to identify the actual pacemaker cell membrane properties modulated by SP, DAMGO, and temperature, it is relevant that several types of pacemaker neurons have been previously described in the respiratory network of the newborn rodent [8, 9] and have also been found recently to exist in the embryo (Chevalier et al., in preparation). This heterogeneity derives mainly from differences in the membrane conductances activated during the pacemakers' bursting activity. Therefore, fully understanding the mechanisms underlying a given neuromodulator's action would require testing for its possible differential effects on distinct pacemaker subtypes through specific actions on different membrane conductance.

Another interesting aspect that we did not test is the possibility that preBötC pacemaker neurons are individually sensitive to multiple modulators that tune respiratory network function through simultaneous diverse and even opposing effects. In fact, in order to avoid nonspecific effects, we deliberately chose to apply only one modulator/temperature increase to each recorded pacemaker cell. The possibility that a given pacemaker neuron might be coregulated by several modulators would require it to possess the appropriate assemblage of receptors and signal transducing pathways. However, the physiological relevance of such a complex process would perhaps attribute the small subpopulation of pacemaker neurons with an overly predominant role in both generating and regulating the rhythmic activity of the preBötC network. Nonetheless, the postnatal respiratory network in rodents is known to be targeted by a variety of modulators [10], including 5HT, acetylcholine, and norepinephrine which have been shown to influence the respiratory network via at least (but not exclusively) an action on pacemaker neurons [12, 15, 16]. A further consideration is that some modulators might be released by the same input terminals, as has been proposed for raphe nuclear projections that can corelease both Substance P and serotonin [25]. Additional experiments are thus required to determine whether such comodulatory influences on preBötC pacemakers are also already effective at prenatal stages. In any case, the modulatory control of the respiratory rhythm is likely to rely on a subtle balance between the influences of different extrinsic substances acting conjointly and possibly by targeting the same neuronal population(s).

The preBötC complex is also interconnected with various other regions of the central nervous system [26], including a second oscillatory network, the parafacial respiratory group (RTN/pFRG), that contributes to generating the overall respiratory command [26–28]. Thus, respiratory rhythm modulation might also rely on a regulation of the activity of this second oscillator and/or of the functional interconnections between the two preBötC and RTN/pFRG oscillators. This renders respiratory rhythm generation potentially even more flexible, especially since it is already known that the two oscillator half centers exhibit diverse sensitivities to the same modulatory agent. For example, while both networks are known to strongly express the NK1 receptor [11, 19, 28], supporting the finding that their function is influenced by Substance P with an excitatory action in both cases, it has also been demonstrated that the preBötC network is alone sensitive to DAMGO, while rhythmogenesis by the RTN/pFRG network is unaffected by the opioid peptide agonist [27, 29, 30]. This selective modulation is particularly important at the time of birth when the maternal release of endogenous opioids causes a depression of preBötC network activity as the baby is born [31–33]. Although this opioid-induced depression could lead to prolonged apnea if not counteracted, excitatory inputs from the unmodulated, still active RTN/pFRG oscillator to the preBötC network might facilitate the rapid reestablishment of inspiratory activity required for survival immediately following birth. This example of the wide functional flexibility of the respiratory central command, while possessing the capability to remain stable and compatible with survival, thus illustrates the need for highly regulated and target-specific influences of the network's different modulatory input pathways. In this context, it is also important to remember that other components of the overall respiratory system may be influenced by neuromodulators, including respiratory motoneurons, muscles, and sensory afferent signaling from the lungs.

In response to a temperature increase, mammals augment their respiratory frequency in order to dissipate heat and decrease body temperature. The present data indicate that this response mechanism is already established in the respiratory network of embryos and, furthermore, is a property of the respiratory pacemaker neurons themselves. Here again, however, the increase in pacemaker cell activity with augmented temperature is unlikely to be solely responsible for the frequency increase observed at the network level. For instance, it is known that a temperature elevation also induces an enhancement of synaptic signaling within respiratory circuitry, with both excitatory and inhibitory synapses being affected [34]. In addition, augmenting temperature also induces changes in the amplitude and duration of inspiratory network bursting [24, 35]. Consequently, intercellular communication and bursting strength can be modified in hyperthermic conditions with resultant modifications in overall network function. We further show here that increasing temperature elicits divergent effects on the subpopulation of respiratory pacemaker neurons. Thus, the combination of these diverse cellular and synaptic influences will together contribute to the respiratory network's response to elevated temperature, with the preBötC pacemaker neurons probably playing a significant role in governing network rhythmogenic frequency. Obviously, our in vitro conditions are substantially different from those in vivo, where multiple factors come into play in the control of respiration as a function of body and brain temperature. So even if our present findings show that respiratory pacemaker neurons might be significantly involved in the respiratory response to temperature changes, other structures are also very likely to play important roles, such as the hypothalamus, vagal feedback, and peripheral and central chemosensitive structures [36–38].

## 5. Conclusion

The respiratory rhythm can be modulated by various internal and external cues. Neuromodulatory processes not only serve the normal stable function of the network but also enable the respiratory command to adapt to changing functional requirements. Our present data indicate that (1) the neuromodulatory processes targeting the respiratory central command are already functional at prenatal stages and (2) that respiratory pacemaker neurons play an important role in mediating these effects. Perturbations in the prenatal development of such modulatory actions might therefore be responsible for inappropriate postnatal breathing behavior, possibly leading to respiratory deficits with potential dramatic consequences as found in several human diseases. Therefore, a better understanding of these regulating processes might have clinical relevance, such as for understanding the Rett and Prader-Willy syndromes or Sudden Infant Death Syndrome, where abnormalities in respiratory neuromodulation mechanisms and temperature control are suspected [39–46].
